# Sofosbuvir as a potential alternative to treat the SARS-CoV-2 epidemic

**DOI:** 10.1038/s41598-020-66440-9

**Published:** 2020-06-09

**Authors:** Rodrigo Jácome, José Alberto Campillo-Balderas, Samuel Ponce de León, Arturo Becerra, Antonio Lazcano

**Affiliations:** 1 Facultad de Ciencias, Universidad Nacional Autónoma de México, Mexico City, Mexico; 2 Programa Universitario de Investigación en Salud, Universidad Nacional Autónoma de México, Mexico City, Mexico; 3 El Colegio Nacional, Mexico City, Mexico

**Keywords:** Molecular medicine, Virology

## Abstract

As of today, there is no antiviral for the treatment of the SARS-CoV-2 infection, and the development of a vaccine might take several months or even years. The structural superposition of the hepatitis C virus polymerase bound to sofosbuvir, a nucleoside analog antiviral approved for hepatitis C virus infections, with the SARS-CoV polymerase shows that the residues that bind to the drug are present in the latter. Moreover, a multiple alignment of several SARS-CoV-2, SARS and MERS-related coronaviruses polymerases shows that these residues are conserved in all these viruses, opening the possibility to use sofosbuvir against these highly infectious pathogens.

## Introduction

A rapid response to a cluster of patients affected with pneumonia of unknown cause in Wuhan, China in December 2019, led to the identification, isolation and sequencing of the SARS-CoV-2. This virus belongs to the genus *Betacoronavirus* (family *Coronaviridae*), which also includes the SARS and MERS-CoVs, which caused epidemics in 2002–2003 and 2012, respectively. Infections by these viruses had higher mortality rates compared to the current COVID-19 outbreak: 9.5%, 34%, and 6.7%, respectively^1–3^.

In recent years we have witnessed the outbreaks of other “emergent” RNA viruses, including the influenza A(H1N1)pdm09 in 2009, ebola in 2014 and 2018–2019, and zika in 2016, in addition to the endemic dengue and yellow fever viral outbursts, which annually infect hundreds of thousands of patients in tropical regions^4^. Combined with their high mutation rates, large population sizes and fast replicative cycles, RNA viral populations quickly explore a vast number of mutational landscapes, which can lead to the emergence of new infectious viruses in humans or viruses with different pathogenic properties. In contrast with other RNA viruses, coronaviruses and other families of the *Nidovirales* order encode for a 3′-5′ exoribonuclease (ExoN) with proofreading activity (nsp14), which diminishes their mutation rate, and is one of the key factors that explains why they are endowed with the longest linear genomes in the RNA virosphere^5^.

As of today, there are no broad-spectrum antivirals available to treat the vast majority of emergent RNA viral infections. This is due to the extreme variability of RNA viral proteomes and the absence of conserved therapeutic targets at which antivirals could be aimed. Currently, the WHO is undertaking the “Solidarity clinical trial for COVID-19 treatments”, a global effort aimed at discovering an efficient treatment against the COVID-19 among those pharmacological resources that have proven to be effective *in vitro* or *in vivo* against SARS-CoV-2 and/or related viruses such as SARS and MERS coronaviruses^6^. The drugs being repurposed in this global effort include HIV-1 protease inhibitors Lopinavir/Ritonavir; Interferon β-1a; the anti-malarial hydroxychloroquine/chloroquine as viral entry inhibitors, and viral RdRp inhibitor Remdesivir. Furthermore, over 950 clinical trials worldwide are registered in the WHO platform as of April 17^th^, 2020, and results from some of them should be available soon.

**Figure 1:**
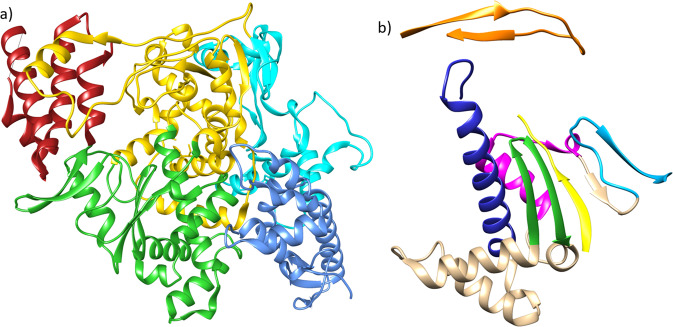
Three-dimensional structure of the SARS-CoV RNA-dependent RNA-polymerase (nsp12) and its palm subdomain. (**a**) The RdRp subdomains are colored as follows: thumb – red; palm – green; fingers – yellow; nidovirus RdRp-associated nucleotidyl transferase (NIRAN) domain – blue; interface – cyan. (**b**) The conserved structural motifs within the palm subdomain and conforming the active site are colored as follows: motif F – orange; motif A – yellow; motif B – blue; motif C – green; motif D – magenta; motif E – cyan.

The most highly conserved protein in all known RNA viruses is the viral monomeric RdRp. The coronavirus replication machinery is a large multi-subunit complex; however, the polymerase domain (nsp12) has the characteristic right-hand shape with fingers, thumb and palm subdomains, and the six conserved structural motifs (Fig. 1)^7^. Structural and phylogenetic analysis indicate that all known viral RdRps are monophyletic and preserve a high degree of structural conservation, in which key residues within six conserved structural motifs partake in the correct nucleotide recognition and incorporation^8^. Nowadays, there are several drugs that bind to the RdRp active site and that have been approved to treat other RNA viral diseases, including Favipiravir^9^ and Remdesivir^10^. This latter is an adenosine analogue, which has been shown to be efficacious preventing different coronaviral infections in mice, and viral populations lacking the ExoN activity are more sensitive to the drug^11^. Recently, this drug proved to be effective blocking SARS-CoV-2 infection *in vitro*^12^.

Sofosbuvir (SOF) is a nucleotide analogue targeted against the HCV polymerase, NS5B. The structure of HCV bound to SOF^13^ reveals that the drug binds to the active site and is incorporated into the nascent strand preventing the addition of the next nucleotide. The residues that participate in SOF binding include motif A’s D225, motif B’s S282, T287, and N291 (the latter binds to the SOF 2′-F), motif F’s K141 and R158, plus motif A’s and C’s universally conserved aspartates that coordinate the metal ions^13^. Previous work has shown that SOF has *in vitro* and/or *in vivo* antiviral activity against other Flaviviruses, i.e. Dengue, Zika, and the West Nile Virus^14–16^. The RdRp structural conservation extends beyond the *Flaviviridae* members and includes all known RNA viruses^8^.

## Results and discussion

**Figure 2:**
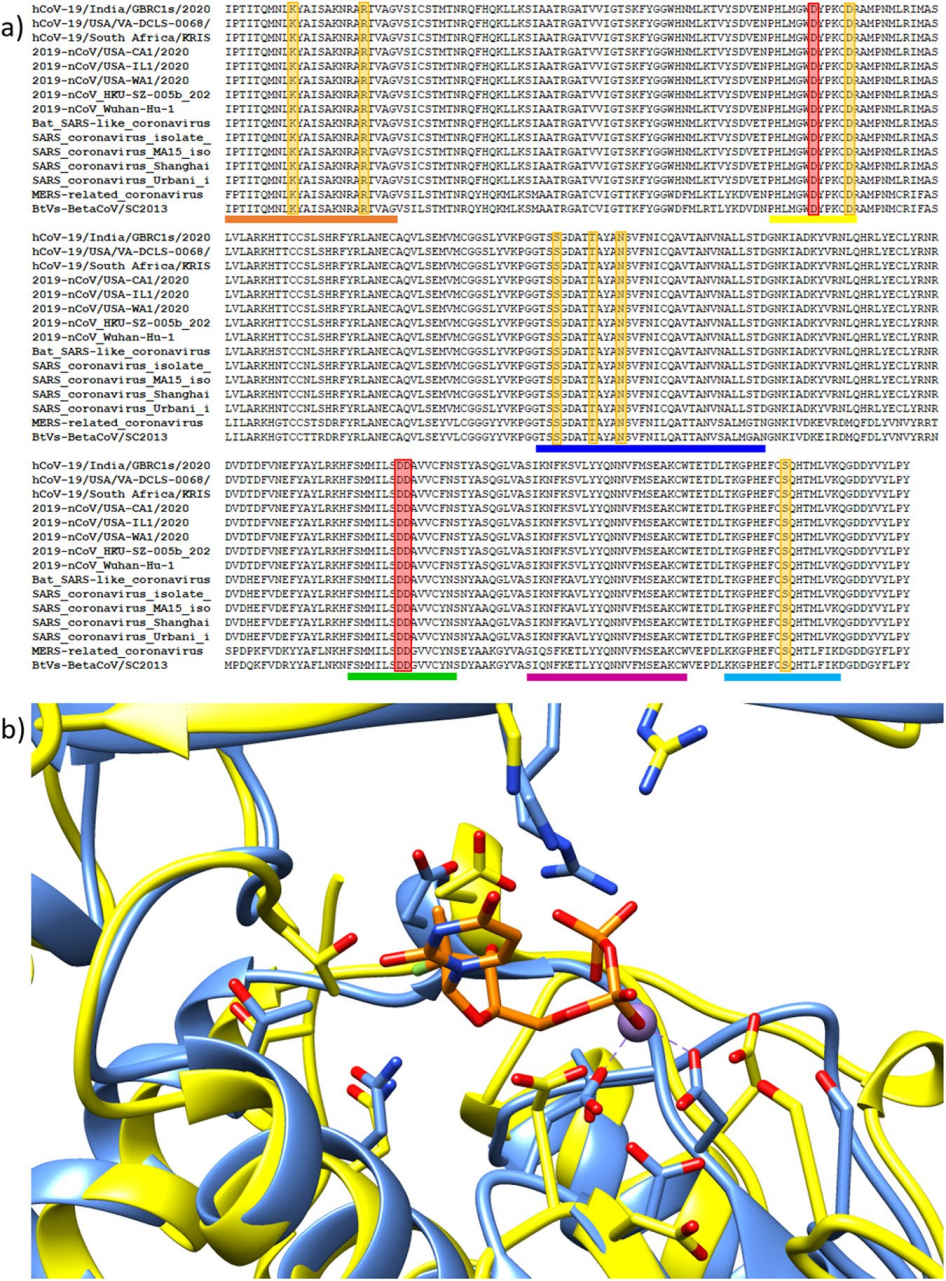
Conservation of the Sofosbuvir binding residues in members of the *Betacoronavirus* genus and structural superposition of SARS-Coronavirus nsp12 with hepatitis C virus NS5B bound to Sofosbuvir. (**a**) Multiple alignment of SARS-CoV-2 nsp12 and other coronaviruses including SARS-CoV and MERS-related coronavirus. The colored lines below the alignment mark the different structural motifs and are the same as Fig. 1; the residues that partake in Sofosbuvir binding are highlighted in orange, whereas the catalytic aspartates are highlighted in red. (**b**) The structural superposition of the two polymerases (HCV NS5B is colored blue; SARS-CoV nsp12 is colored yellow) shows the high degree of conservation in the active site. The sidechains of the residues partaking in Sofosbuvir binding are shown, and sofosbuvir is colored orange.

The multiple alignment of the SARS-CoV nsp12 sequence with distinct SARS-CoV-2 nsp12 sequences and MERS-related coronaviruses shows that the SOF-binding residues are conserved (Fig. 2a). As expected, even the most recent SARS-CoV-2 sequences present a strict conservation of the polymerase catalytic domain and the binding residues (Fig. 2a). A structural superposition of the SARS-coronavirus nsp12 with HCV NS5B bound to SOF shows that the inhibitor can be modeled into the nsp12’s active site without any steric hindrances, and that the residues that partake in SOF binding are well conserved in the SARS-coronavirus active site (Fig. 2b). As observed in Fig. 2b, some of the residues’ side-chains involved in SOF-binding and catalytic activities have different conformations in the two polymerases. This might be explained by the fact that the HCV NS5B is in an active conformation, whereas the SARS-CoV nsp12 is in its apo-form. Neither the results presented here nor a recently published independent model^7^ support the possibility that the conserved T680 found in coronaviruses but absent in NS5B is required for SOF binding to the RdRp active site. Detailed characterization of the interaction between T680 and SOF must await experimental analysis and/or the availability of a three-dimensional structure of SARS-CoV-2 nsp12 bound to SOF. While this manuscript was under review, very similar conclusions have been reported by Elfiky^17^. The work built an homology-based model of the SARS-CoV-2 nsp12 and performed molecular docking experiments to test if Sofosbuvir, as well as other nucleoside analogs, might be effective against the virus, yielding very promising results.

With the number of COVID-19 cases surpassing the 2 million and fatalities reaching over 160 000 deaths, the resources and the options being tested against the virus are increasing in a parallel way, with serious proposals of testing stem-cells and traditional Chinese medicines as antivirals^18^. Considering our *in silico* results and the work by Elfiky^17^, added to the fact that SOF has already been approved as a standard treatment and has a well-known safety profile^19^, it would be interesting to develop *in vitro* experiments to test its efficacy against the SARS-CoV-2 as well as its minimal inhibitory concentration as a step towards later testing in a clinical setting. Although SOF has a high barrier to resistance, several point mutations have been identified in the HCV NS5B that confer resistance to this drug in clinical settings, which may occur in the SARS-CoV-2 nsp12^20^. Furthermore, since the presence of the proofreading enzyme has been shown to interfere with the antiviral activity of nucleoside analogs by their removal from the nascent chain in coronaviral infections^21^, as suggested by the use of Remdesivir-based therapies^11^, it can be speculated that a SOF regime in high concentrations might circumvent the action of the ExoN and prevent the removal of the drug. The SARS-CoV-2 RdRp inhibition might represent a key step towards a control of the COVID-19 pandemic; nevertheless, the two latter aspects and previous RNA viral infections suggest that a multitargeted approach, in which different drugs target different viral proteins, might be the ultimate strategy.

From an evolutionary perspective, the highly conserved palm subdomain of viral RdRps might be the RNA viral Achilles heel. The availability of additional tertiary structures can help to understand the differences and the relatedness between viral RdRps, which, in turn, may lead to more alternatives towards the development of broad spectrum antivirals.

## Materials and Methods

### Coronavirus nsp12 multiple alignment

From the orf1ab polyprotein (Wuhan seafood market pneumonia virus isolate Wuhan-Hu-1) deposited in the NCBI (NC_045512.2), we selected the nsp12 RNA-dependent RNA polymerase sequence (protein id. YP_009725307.1). A BLASTp was performed and representative sequences of current SARS-CoV-2, SARS- and MERS-related coronaviruses as well as two bat betacoronaviruses were downloaded. In order to analyze the possibility that mutations might have emerged during the four months of the COVID-19 pandemic, we downloaded sequences from samples collected in April from the GISAID platform^22^. The multiple alignment was performed in MEGA X^23^ with the ClustalW algorithm using default parameters. The multiple alignment was edited in BioEdit^24^, only the residues corresponding to the conserved structural motifs A-F are shown.

### Structural superposition of hepatitis C virus and SARS-CoV polymerases

The structural superposition of the hepatitis C virus polymerase NS5B bound to Sofosbuvir [PDB ID: 4WTG^13^] with the SARS-coronavirus nsp12 [PDB ID: 6NUR^7^] was performed in Chimera 1.13^25^ using the MatchMaker^26^ algorithm with default parameters. The tertiary structures were edited and depicted with Chimera 1.13^25^.
